# Supplementary material for: Modelling Protein Plasticity: The Example of Frataxin and Its Variants
Source: Molecules. 2022 Mar 17;27(6):1955. doi: 10.3390/molecules27061955 (PMC8950120; doi:10.3390/molecules27061955)
Supplement: Supplementary file 1 [file molecules-27-01955-s001.zip › molecules-1601093-supplementary.pdf]

Modelling proteins' plasticity:
The example of frataxin and its variants.
Supplementary Materials

S. Botticelli, G. La Penna, G. Nobili, G.C. Rossi, F. Stellato, S. Morante

- Table S 1 - Table indicating the HGVS variant corresponding to the labels used in this work.
- Fig. S 1 - Figure describing the change of free energy with FXN unfolding.
- Fig. S 2 - Figure describing the change of approximated free energy with FXN unfolding.
- Fig. S 3 - Figure describing the change of SASA with FXN unfolding for different groups of atoms in the protein.

Table S1: Variants indicated in the HGVS format corresponding to the variants used in the manuscript

HGVS format	Abbreviated	Abbreviated in text and figures
NM_000144.4:c.311A>G	p.D104G	D104G
NM_000144.4:c.320C>T	p.A107V	A107V
NM_000144.4:c.327T>A	p.F109L	F109L
NM_000144.4:c.368A>C	p.Y123S	Y123S
NM_000144.4:c.482G>T	p.S161I	S161I
NM_000144.4:c.519G>T	p.W173C	W173C
NM_000144.4:c.542C>T	p.S181F	S181F
NM_000144.4:c.605C>T	p.S202F	S202F

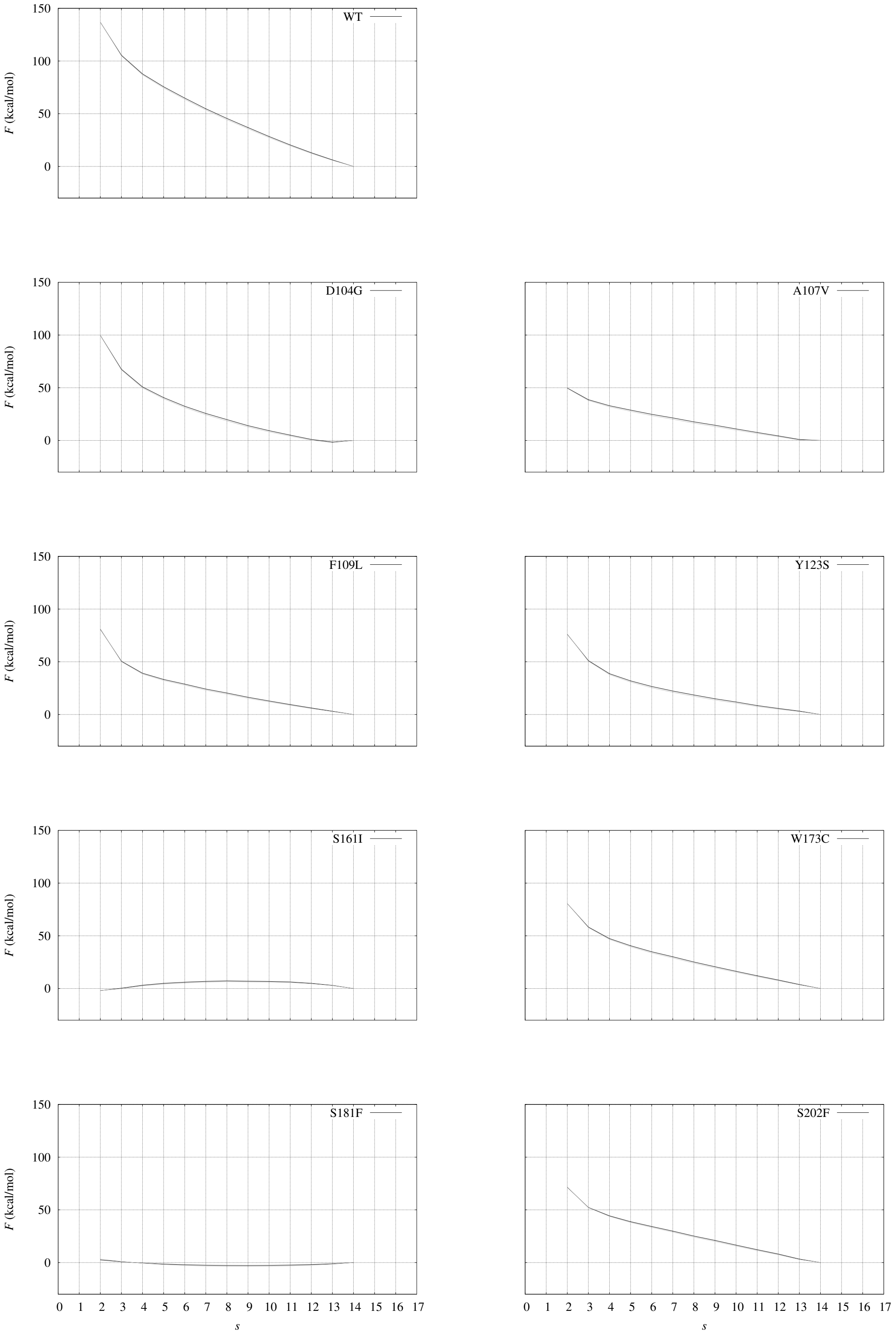

Figure S1: Free energy as a function of the collective variable s for native (WT) and mutated sequences (variants are indicated in each panel). The meta-statistics is that in Eq. 2. T in Eq. 10 is 300 K (black curve) and 400 K (gray curve), but curves are overlapping.

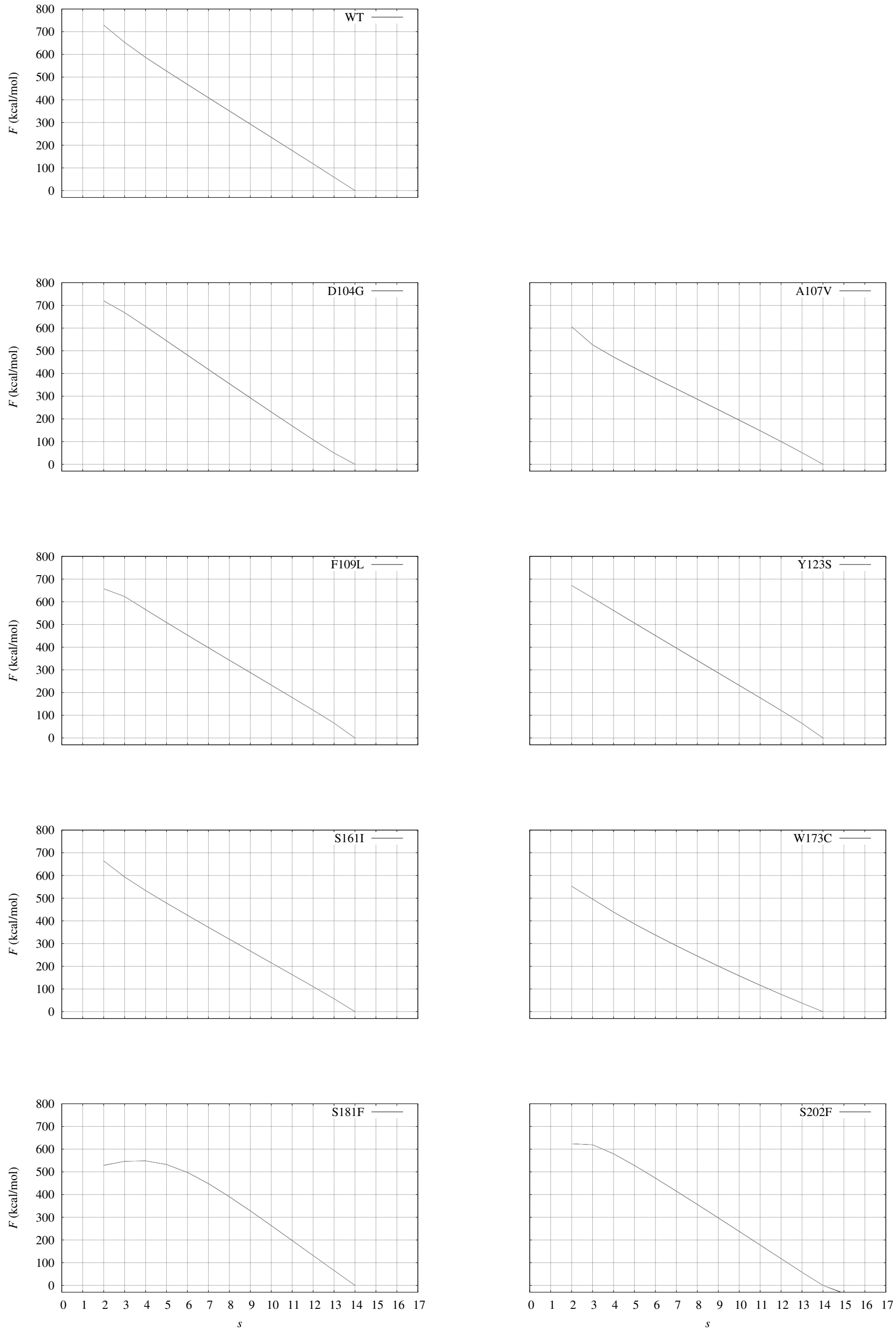

Figure S2: Same as for Fig. S 1 using mean-field solvation free energy (Eq. 12) in Eq. 10.

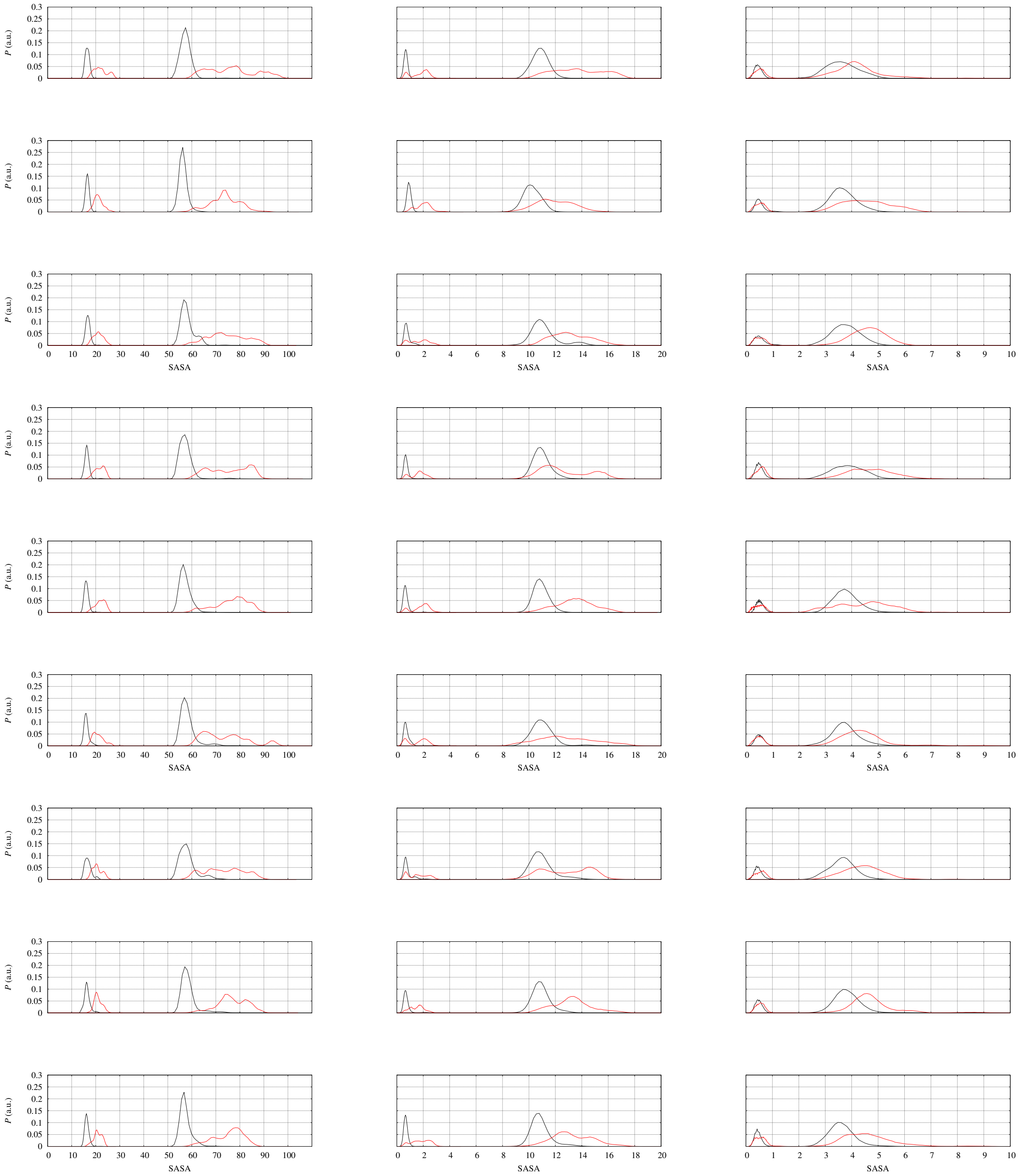

Figure S3: Distribution of relative SASA (SASA divided for the maximal SASA of the same group) for protein backbone (thick curve) and sidechains (thin curve), by imposing $\langle \mathrm{CV} \rangle$ =14 (black) $\langle \mathrm{CV} \rangle$ =2 (red). From top to bottom - WT, D104G, A107V; from left to right - all residues, $\alpha 1$ (92-114), $\alpha 2$ (182-193).
